# 2-{[2-(2-Hy­droxy-3-meth­oxy­benzyl­idene)hydrazin-1-yl­idene]meth­yl}-6-meth­oxy­phenol

**DOI:** 10.1107/S1600536811036816

**Published:** 2011-09-30

**Authors:** Rong Lu, Wenxia Wang, Xingqiang Lü, Shunsheng Zhao

**Affiliations:** a College of Chemical Engineering, Northwest University, Xi’an 710069, Shaanxi, People’s Republic of China; b College of Chemistry and Chemical Engineering, Xian University of Science and Technology, Xi’an 710054, Shaanxi, People’s Republic of China

## Abstract

The title compound, C_16_H_16_N_2_O_4_, was obtained from the reaction of hydrazine hydrate and *o*-vanilin in absolute ethanol. The mol­ecule is almost planar (except for the methyl H atoms), with a mean deviation from the plane of 0.0259 Å. The mol­ecular structure also exhibits an approximate non-crystallographic twofold axis. Intra­molecular O—H⋯N hydrogen bonds occur. In the crystal, inter­molecular C—H⋯O hydrogen bonds generate mol­ecular zigzag sheets. The sheets stack through C—H⋯π inter­actions, leading to a three-dimensional-network.

## Related literature

For the properties and applications of the title compound or similar structural compounds and their metal complexes, see: Lin *et al.* (2009 ▶); Davidson *et al.* (2006 ▶); Lin & Zeng (2006 ▶). 
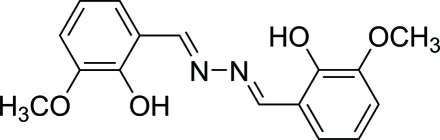


## Experimental

### 

#### Crystal data

C_16_H_16_N_2_O_4_
*M* *_r_* = 300.31
Monoclinic, 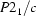

*a* = 6.3095 (14) Å
*b* = 17.405 (4) Å
*c* = 13.606 (3) Å
β = 95.590 (4)°
*V* = 1487.0 (6) Å^3^
*Z* = 4
Mo *K*α radiation
μ = 0.10 mm^−1^
*T* = 296 K
0.25 × 0.20 × 0.18 mm

#### Data collection

Bruker SMART 1K CCD area-detector diffractometer
Absorption correction: multi-scan (*SADABS*; Sheldrick, 2004 ▶) *T*_min_ = 0.858, *T*_max_ = 1.000
7393 measured reflections
2648 independent reflections
1133 reflections with *I* > 2σ(*I*)
*R*_int_ = 0.052

#### Refinement

*R*[*F* ^2^ > 2σ(*F* ^2^)] = 0.061
*wR*(*F* ^2^) = 0.186
*S* = 1.07
2648 reflections
208 parameters
H atoms treated by a mixture of independent and constrained refinement
Δρ_max_ = 0.23 e Å^−3^
Δρ_min_ = −0.23 e Å^−3^

### 

Data collection: *SMART* (Bruker, 2001 ▶); cell refinement: *SAINT* (Bruker, 2001 ▶); data reduction: *SAINT*; program(s) used to solve structure: *SHELXS97* (Sheldrick, 2008 ▶); program(s) used to refine structure: *SHELXL97* (Sheldrick, 2008 ▶); molecular graphics: *SHELXTL* (Sheldrick, 2008 ▶); software used to prepare material for publication: *SHELXTL*.

## Supplementary Material

Crystal structure: contains datablock(s) I, global. DOI: 10.1107/S1600536811036816/fl2353sup1.cif

Structure factors: contains datablock(s) I. DOI: 10.1107/S1600536811036816/fl2353Isup2.hkl

Supplementary material file. DOI: 10.1107/S1600536811036816/fl2353Isup3.cml

Additional supplementary materials:  crystallographic information; 3D view; checkCIF report

## Figures and Tables

**Table 1:** Hydrogen-bond geometry (Å, °) *Cg*1 and *Cg*2 are the centroids of the C2–C7 and C10–C15 rings, respectively.

*D*—H⋯*A*	*D*—H	H⋯*A*	*D*⋯*A*	*D*—H⋯*A*
O3—H3*A*⋯N2	0.91 (5)	1.82 (5)	2.640 (4)	149 (4)
O2—H2*A*⋯N1	0.88 (4)	1.82 (4)	2.636 (4)	153 (4)
C16—H16*A*⋯O4^i^	0.96	2.55	3.279 (5)	133
C7—H7*A*⋯*Cg*2^ii^	0.93	2.90	3.694 (4)	144
C13—H13*A*⋯*Cg*1^iii^	0.93	2.89	3.717 (4)	149

Symmetry codes: (i) 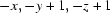; (ii) 
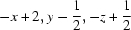
; (iii) 
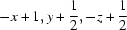
.

**Fig. 1.**
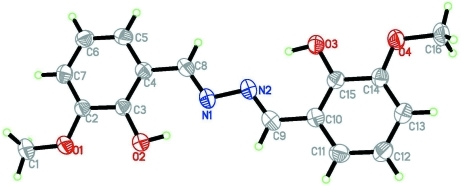
The molecular structure of (I), with atom labels and 30% probability displacement ellipsoids for non-H atoms.

**Fig. 2.**
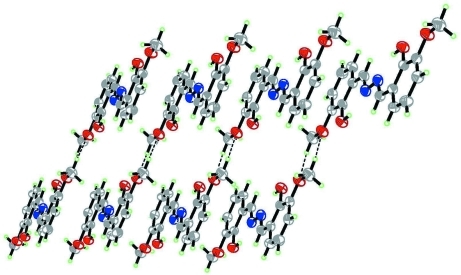
The packing of (I), showing one chain of molecules connected by C—H···O hydrogen bonds (dashed lines). H atoms not involved in hydrogen bonding have been omitted.

**Fig. 3.**
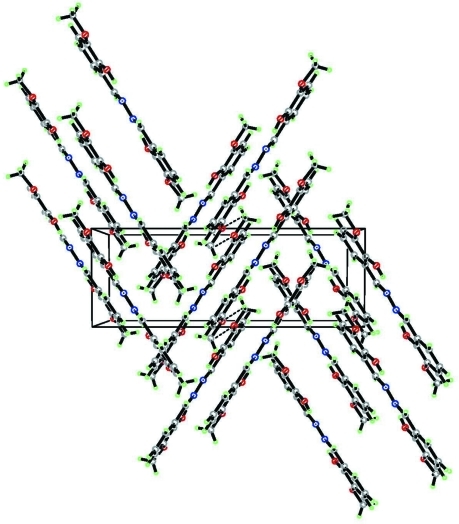
The packing of (I), showing one layer of molecules connected by stacking interaction.

## supplementary crystallographic information

### Comment

The title compound, (I) (Fig. 1), with various chelating atoms, could coordinate with many transition metals (Davidson *et al., 2006*) and lanthanides (Lin and Zeng, 2006; Lin *et al. , 2009*) to form functional complexes. The molecule crystallizes in the monoclinic space group P21/c and appears to be almost completely planar (except for the methyl hydrogen atoms) with a mean deviation from the plane ooff 0.0259 Å. The molecule also exhibits a non-crystallographic 2-fold axis. There are intramolecular O—H···N hydrogen bonds, intermolecular C—H···O hydrogen bonds and C—H···π hydrogen bonds. Molecules are linked by the C—H···O hydrogen bonds, generating molecular zigzag sheets, as shown in Fig. 2. The C—H···π hydrogen bonds and stacking interaction of these sheets leads to a three-dimensional-network. (Fig. 3).

### Experimental

The title compound was obtained from the reaction of hydrazine hydrate and *o*-vanilin in absolute ethanol. Hydrazine hydrate (500 mg, 10 mmol) was added to a solution of *o*-vanilin (3.04 g, 20 mmol) in absolute ethanol (200 ml) and heated to reflux for 2 h.  The resulting solution was allowed to evaporate at rt to give a yellow crystal, which was collected by filtration and dried under vacumn;; yield 89.3%. The single-crystal of the title compound suitble for X-ray diffraction was obtained by recrystalization from absolute ethanol.

### Refinement

H atoms bonded to O atoms were refined isotropically without restraints, and with *U*_iso_(H) = 1.2*U*_eq_(O). Other H atoms were positioned geometrically and refined using a riding model with C—H = 0.95–0.99 Å and with *U*_iso_(H) = 1.2 (1.5 for methyl groups) times *U*_eq_(C).

### Crystal data

**Table:**

C_16_H_16_N_2_O_4_	*F*(000) = 632
*M**_r_* = 300.31	*D*_x_ = 1.341 Mg m^−^^3^
Monoclinic, *P*2_1_/*c*	Mo *K*α radiation, λ = 0.71073 Å
*a* = 6.3095 (14) Å	Cell parameters from 8451 reflections
*b* = 17.405 (4) Å	θ = 1.9–26.6°
*c* = 13.606 (3) Å	µ = 0.10 mm^−^^1^
β = 95.590 (4)°	*T* = 296 K
*V* = 1487.0 (6) Å^3^	Block, yellow
*Z* = 4	0.25 × 0.20 × 0.18 mm

### Data collection

**Table:**

Bruker SMART 1K CCD area-detector diffractometer	2648 independent reflections
Radiation source: fine-focus sealed tube	1133 reflections with *I* > 2σ(*I*)
graphite	*R*_int_ = 0.052
thin–slice ω scans	θ_max_ = 25.1°, θ_min_ = 1.9°
Absorption correction: multi-scan (*SADABS*; Sheldrick, 2004)	*h* = −6→7
*T*_min_ = 0.858, *T*_max_ = 1.000	*k* = −18→20
7393 measured reflections	*l* = −15→16

### Refinement

**Table:**

Refinement on *F*^2^	Primary atom site location: structure-invariant direct methods
Least-squares matrix: full	Secondary atom site location: difference Fourier map
*R*[*F*^2^ > 2σ(*F*^2^)] = 0.061	Hydrogen site location: inferred from neighbouring sites
*wR*(*F*^2^) = 0.186	H atoms treated by a mixture of independent and constrained refinement
*S* = 1.07	*w* = 1/[σ^2^(*F*_o_^2^) + (0.0677*P*)^2^] where *P* = (*F*_o_^2^ + 2*F*_c_^2^)/3
2648 reflections	(Δ/σ)_max_ < 0.001
208 parameters	Δρ_max_ = 0.23 e Å^−^^3^
0 restraints	Δρ_min_ = −0.23 e Å^−^^3^

### Special details

**Table:**

Geometry. All e.s.d.'s (except the e.s.d. in the dihedral angle between two l.s. planes) are estimated using the full covariance matrix. The cell e.s.d.'s are taken into account individually in the estimation of e.s.d.'s in distances, angles and torsion angles; correlations between e.s.d.'s in cell parameters are only used when they are defined by crystal symmetry. An approximate (isotropic) treatment of cell e.s.d.'s is used for estimating e.s.d.'s involving l.s. planes.
Refinement. Refinement of *F*^2^ against ALL reflections. The weighted *R*-factor *wR* and goodness of fit *S* are based on *F*^2^, conventional *R*-factors *R* are based on *F*, with *F* set to zero for negative *F*^2^. The threshold expression of *F*^2^ > σ(*F*^2^) is used only for calculating *R*-factors(gt) *etc*. and is not relevant to the choice of reflections for refinement. *R*-factors based on *F*^2^ are statistically about twice as large as those based on *F*, and *R*- factors based on ALL data will be even larger.

### Fractional atomic coordinates and isotropic or equivalent isotropic displacement parameters (Å^2^)

**Table:**

	*x*	*y*	*z*	*U*_iso_*/*U*_eq_	
O2	1.1229 (5)	0.30718 (16)	0.11097 (19)	0.0657 (8)	
O3	0.3751 (4)	0.44682 (15)	0.36771 (19)	0.0659 (8)	
N2	0.6605 (5)	0.39741 (18)	0.2529 (2)	0.0603 (9)	
N1	0.8399 (5)	0.35841 (17)	0.2254 (2)	0.0593 (9)	
C4	1.1395 (6)	0.28216 (19)	0.2861 (3)	0.0529 (10)	
O1	1.4697 (4)	0.23030 (15)	0.09342 (18)	0.0708 (8)	
C2	1.4056 (6)	0.2325 (2)	0.1861 (3)	0.0533 (10)	
O4	0.0317 (4)	0.52385 (14)	0.38754 (18)	0.0696 (8)	
C14	0.0966 (6)	0.5236 (2)	0.2954 (3)	0.0555 (10)	
C3	1.2189 (6)	0.27497 (19)	0.1943 (3)	0.0513 (9)	
C15	0.2831 (6)	0.4809 (2)	0.2850 (3)	0.0532 (10)	
C13	−0.0034 (6)	0.5612 (2)	0.2144 (3)	0.0628 (11)	
H13A	−0.1247	0.5904	0.2210	0.075*	
C10	0.3625 (6)	0.4756 (2)	0.1936 (3)	0.0570 (10)	
C8	0.9494 (6)	0.3256 (2)	0.2985 (3)	0.0585 (11)	
H8A	0.9043	0.3300	0.3613	0.070*	
C11	0.2542 (7)	0.5129 (2)	0.1132 (3)	0.0702 (12)	
H11A	0.3043	0.5086	0.0514	0.084*	
C5	1.2488 (6)	0.2463 (2)	0.3685 (3)	0.0638 (11)	
H5A	1.1981	0.2512	0.4301	0.077*	
C7	1.5070 (6)	0.1973 (2)	0.2683 (3)	0.0624 (11)	
H7A	1.6292	0.1684	0.2627	0.075*	
C9	0.5516 (6)	0.4314 (2)	0.1809 (3)	0.0633 (11)	
H9A	0.5963	0.4273	0.1179	0.076*	
C6	1.4279 (7)	0.2047 (2)	0.3592 (3)	0.0674 (11)	
H6A	1.4980	0.1810	0.4144	0.081*	
C16	−0.1522 (6)	0.5682 (2)	0.4042 (3)	0.0783 (14)	
H16A	−0.1799	0.5635	0.4721	0.117*	
H16B	−0.2728	0.5497	0.3624	0.117*	
H16C	−0.1274	0.6211	0.3893	0.117*	
C12	0.0759 (7)	0.5556 (2)	0.1232 (3)	0.0731 (12)	
H12A	0.0079	0.5810	0.0688	0.088*	
C1	1.6443 (6)	0.1816 (2)	0.0767 (3)	0.0817 (14)	
H1B	1.6730	0.1854	0.0089	0.123*	
H1C	1.6095	0.1294	0.0914	0.123*	
H1D	1.7680	0.1972	0.1186	0.123*	
H3A	0.490 (8)	0.421 (3)	0.349 (4)	0.128 (19)*	
H2A	1.007 (7)	0.328 (2)	0.131 (3)	0.096 (16)*	

### Atomic displacement parameters (Å^2^)

**Table:**

	*U*^11^	*U*^22^	*U*^33^	*U*^12^	*U*^13^	*U*^23^
O2	0.0594 (19)	0.080 (2)	0.0581 (18)	0.0130 (15)	0.0097 (15)	0.0022 (14)
O3	0.0600 (19)	0.0752 (19)	0.0638 (19)	0.0177 (15)	0.0130 (15)	0.0115 (14)
N2	0.052 (2)	0.060 (2)	0.071 (2)	0.0020 (16)	0.0164 (17)	−0.0059 (17)
N1	0.049 (2)	0.060 (2)	0.071 (2)	0.0004 (16)	0.0146 (18)	−0.0089 (17)
C4	0.055 (2)	0.051 (2)	0.053 (2)	−0.0052 (18)	0.0082 (19)	−0.0062 (18)
O1	0.0661 (18)	0.0868 (19)	0.0619 (18)	0.0217 (15)	0.0187 (14)	0.0032 (14)
C2	0.050 (2)	0.055 (2)	0.055 (2)	0.0014 (19)	0.0069 (19)	−0.0041 (18)
O4	0.0630 (18)	0.0796 (19)	0.0691 (18)	0.0180 (15)	0.0214 (14)	0.0114 (14)
C14	0.049 (2)	0.055 (2)	0.063 (3)	−0.0023 (19)	0.008 (2)	0.0028 (19)
C3	0.050 (2)	0.052 (2)	0.052 (2)	−0.0001 (19)	0.0042 (18)	−0.0021 (18)
C15	0.052 (2)	0.051 (2)	0.056 (2)	−0.0026 (19)	0.0052 (19)	0.0064 (18)
C13	0.056 (3)	0.059 (3)	0.073 (3)	0.0026 (19)	0.003 (2)	0.007 (2)
C10	0.054 (2)	0.054 (2)	0.064 (3)	−0.0039 (19)	0.009 (2)	−0.0012 (19)
C8	0.056 (3)	0.059 (2)	0.062 (3)	−0.006 (2)	0.015 (2)	−0.011 (2)
C11	0.071 (3)	0.081 (3)	0.059 (3)	−0.003 (2)	0.010 (2)	0.002 (2)
C5	0.071 (3)	0.068 (3)	0.053 (3)	−0.007 (2)	0.008 (2)	−0.007 (2)
C7	0.058 (3)	0.059 (3)	0.070 (3)	0.008 (2)	0.005 (2)	0.000 (2)
C9	0.065 (3)	0.060 (3)	0.067 (3)	−0.005 (2)	0.020 (2)	−0.008 (2)
C6	0.074 (3)	0.068 (3)	0.059 (3)	0.010 (2)	0.001 (2)	0.0008 (19)
C16	0.054 (3)	0.093 (3)	0.091 (3)	0.018 (2)	0.021 (2)	0.006 (2)
C12	0.067 (3)	0.079 (3)	0.071 (3)	0.005 (2)	−0.005 (2)	0.009 (2)
C1	0.065 (3)	0.098 (3)	0.085 (3)	0.019 (3)	0.023 (2)	−0.004 (2)

### Geometric parameters (Å, °)

**Table:**

O2—C3	1.354 (4)	C13—H13A	0.9300
O2—H2A	0.88 (4)	C10—C11	1.392 (5)
O3—C15	1.352 (4)	C10—C9	1.445 (5)
O3—H3A	0.91 (5)	C8—H8A	0.9300
N2—C9	1.285 (4)	C11—C12	1.366 (5)
N2—N1	1.402 (4)	C11—H11A	0.9300
N1—C8	1.288 (4)	C5—C6	1.359 (5)
C4—C3	1.396 (4)	C5—H5A	0.9300
C4—C5	1.404 (5)	C7—C6	1.384 (5)
C4—C8	1.441 (5)	C7—H7A	0.9300
O1—C2	1.361 (4)	C9—H9A	0.9300
O1—C1	1.426 (4)	C6—H6A	0.9300
C2—C7	1.377 (5)	C16—H16A	0.9600
C2—C3	1.404 (5)	C16—H16B	0.9600
O4—C14	1.357 (4)	C16—H16C	0.9600
O4—C16	1.430 (4)	C12—H12A	0.9300
C14—C13	1.380 (5)	C1—H1B	0.9600
C14—C15	1.411 (5)	C1—H1C	0.9600
C15—C10	1.388 (5)	C1—H1D	0.9600
C13—C12	1.385 (5)		
			
C3—O2—H2A	103 (3)	C12—C11—C10	121.4 (4)
C15—O3—H3A	106 (3)	C12—C11—H11A	119.3
C9—N2—N1	113.9 (3)	C10—C11—H11A	119.3
C8—N1—N2	113.3 (3)	C6—C5—C4	120.7 (3)
C3—C4—C5	118.9 (3)	C6—C5—H5A	119.6
C3—C4—C8	121.8 (4)	C4—C5—H5A	119.6
C5—C4—C8	119.3 (3)	C2—C7—C6	120.3 (4)
C2—O1—C1	117.9 (3)	C2—C7—H7A	119.8
O1—C2—C7	125.7 (3)	C6—C7—H7A	119.8
O1—C2—C3	114.6 (3)	N2—C9—C10	122.7 (3)
C7—C2—C3	119.8 (3)	N2—C9—H9A	118.6
C14—O4—C16	118.1 (3)	C10—C9—H9A	118.6
O4—C14—C13	125.5 (3)	C5—C6—C7	120.6 (4)
O4—C14—C15	115.1 (3)	C5—C6—H6A	119.7
C13—C14—C15	119.4 (3)	C7—C6—H6A	119.7
O2—C3—C4	122.8 (3)	O4—C16—H16A	109.5
O2—C3—C2	117.5 (3)	O4—C16—H16B	109.5
C4—C3—C2	119.7 (4)	H16A—C16—H16B	109.5
O3—C15—C10	123.6 (3)	O4—C16—H16C	109.5
O3—C15—C14	116.3 (3)	H16A—C16—H16C	109.5
C10—C15—C14	120.1 (4)	H16B—C16—H16C	109.5
C14—C13—C12	120.3 (4)	C11—C12—C13	120.0 (4)
C14—C13—H13A	119.8	C11—C12—H12A	120.0
C12—C13—H13A	119.8	C13—C12—H12A	120.0
C15—C10—C11	118.7 (4)	O1—C1—H1B	109.5
C15—C10—C9	121.1 (4)	O1—C1—H1C	109.5
C11—C10—C9	120.1 (3)	H1B—C1—H1C	109.5
N1—C8—C4	122.2 (3)	O1—C1—H1D	109.5
N1—C8—H8A	118.9	H1B—C1—H1D	109.5
C4—C8—H8A	118.9	H1C—C1—H1D	109.5

### Hydrogen-bond geometry (Å, °)

**Table:**

Cg1 and Cg2 are the centroids of the C2–C7 and C10–C15 rings, respectively.

**Table:**

*D*—H···*A*	*D*—H	H···*A*	*D*···*A*	*D*—H···*A*
O3—H3A···N2	0.91 (5)	1.82 (5)	2.640 (4)	149 (4)
O2—H2A···N1	0.88 (4)	1.82 (4)	2.636 (4)	153 (4)
C16—H16A···O4^i^	0.96	2.55	3.279 (5)	133
C7—H7A···Cg2^ii^	0.93	2.90	3.694 (4)	144
C13—H13A···Cg1^iii^	0.93	2.89	3.717 (4)	149

Symmetry codes: (i) −*x*, −*y*+1, −*z*+1; (ii) −*x*+2, *y*−1/2, −*z*+1/2; (iii) −*x*+1, *y*+1/2, −*z*+1/2.
